# Epidemiology of Geographic Disparities of Myocardial Infarction Among Older Adults in the United States: Analysis of 2000–2017 Medicare Data

**DOI:** 10.3389/fcvm.2021.707102

**Published:** 2021-09-09

**Authors:** Bin Yu, Igor Akushevich, Arseniy P. Yashkin, Julia Kravchenko

**Affiliations:** ^1^Department of Surgery, School of Medicine, Duke University, Durham, NC, United States; ^2^Social Science Research Institute, Duke University, Durham, NC, United States; ^3^Department of Preventive Medicine, School of Health Sciences, Wuhan University, Wuhan, China

**Keywords:** myocardial infarction, life expectancy, geographic disparity, leading and lagging states, mortality, survival

## Abstract

**Background:** There are substantial geographic disparities in the life expectancy (LE) across the U.S. with myocardial infarction (MI) contributing significantly to the differences between the states with highest (leading) and lowest (lagging) LE. This study aimed to systematically investigate the epidemiology of geographic disparities in MI among older adults.

**Methods:** Data on MI outcomes among adults aged 65+ were derived from the Center for Disease Control and Prevention-sponsored Wide-Ranging Online Data for Epidemiologic Research database and a 5% sample of Medicare Beneficiaries for 2000–2017. Death certificate-based mortality from MI as underlying/multiple cause of death (CBM-UCD/CBM-MCD), incidence-based mortality (IBM), incidence, prevalence, prevalence at age 65, and 1-, 3-, and 5-year survival, and remaining LE at age 65 were estimated and compared between the leading and lagging states. Cox model was used to investigate the effect of residence in the lagging states on MI incidence and survival.

**Results:** Between 2000 and 2017, MI mortality was higher in the lagging than in the leading states (per 100,000, CBM-UCD: 236.7–583.7 vs. 128.2–357.6, CBM-MCD: 322.7–707.7 vs. 182.4–437.7, IBM: 1330.5–1518.9 vs. 1003.3–1197.0). Compared to the leading states, lagging states had higher MI incidence (1.1–2.0% vs. 0.9–1.8%), prevalence (10.2–13.1% vs. 8.3–11.9%), pre-existing prevalence (2.5–5.1% vs. 1.4–3.6%), and lower survival (70.4 vs. 77.2% for 1-year, 63.2 vs. 67.2% for 3-year, and 52.1 vs. 58.7% for 5-year), and lower remaining LE at age 65 among MI patients (years, 8.8–10.9 vs. 9.9–12.8). Cox model results showed that the lagging states had greater risk of MI incidence [Adjusted hazards ratio, AHR (95% Confidence Interval, CI): 1.18 (1.16, 1.19)] and death after MI diagnosis [1.22 (1.21, 1.24)]. Study results also showed alarming declines in survival and remaining LE at age 65 among MI patients.

**Conclusion:** There are substantial geographic disparities in MI outcomes, with lagging states having higher MI mortality, incidence, and prevalence, lower survival and remaining LE at age 65. Disparities in MI mortality in a great extent could be due to between-the-state differences in MI incidence, prevalence at age 65 and survival. Observed declines in survival and remaining LE require an urgent analysis of contributing factors that must be addressed.

## Introduction

Myocardial infarction (MI) is a challenging clinical and public health problem among older adults in the United States (U.S.) (1, 2). MI is one of the most important risk factors for heart failure (HF) (3, 4) which contributes substantially to the geographic disparities in life expectancy (LE) (5), that have been observed for decades in the U.S. with the highest LE of 82.0 years in Hawaii and the lowest of 74.9 in Mississippi (data for 2017) (6). The underlying mechanisms of the disparities between the states with the highest and lowest LE (referred to in this text as “leading” and “lagging” states) are complex and not fully understood. Understanding the patterns of MI outcomes in states leading and lagging in LE provides additional opportunities for mitigating both MI and HF disparities.

MI occurs once every 40 seconds in the U.S., with an estimated annual incidence of 605,000 new cases and 200,000 recurrent cases (1, 2), and a prevalence of 3.0% for U.S. adults aged 20 years and older during 2013-2016 (1). MI is more prevalent among older adults with an average age of the first MI being 65.6 years for males and 72.0 years for females (1, 2). MI mortality in the U.S. was 27.0 per 100,000 in 2018 with highest rate being observed in Arkansas and the lowest in Alaska (7). Despite the well-studied sex and racial disparities (1, 8–10), factors contributing to geographic disparities in MI mortality are not fully understood. Due to the great improvements in MI treatment and management (4), a substantial declining trend of MI mortality has been observed in the past decades (1, 11); however, recent studies reported increasing mortality from ST segment Elevation MI (STEMI, one important subtype of MI) (12), mortality in MI patients with comorbidities (13, 14), as well as the post-discharge MI mortality (15, 16).

It is challenging to investigate the underlying mechanisms of the geographic disparities in MI mortality. Possible epidemiologic scenarios explaining the disparities may include the following: regions/states with higher MI mortality may also have (a) a higher MI incidence; (b) poorer survival of MI patients; and (c) higher pre-existing MI prevalence (i.e., at time the older adults are enrolled in the Medicare program) (17). These scenarios may work independently or together in contributing to geographic disparities in MI mortality across the U.S.

In this study, we aimed to investigate the epidemiology of the geographic disparities in MI outcomes (e.g., mortality, incidence, prevalence, survival, remaining LE at age 65) between states leading and lagging by LE, and discuss the underlying scenarios that may explain the disparities in MI mortality by analyzing data from death certificate and a 5% sample of Medicare Beneficiaries. We hypothesize that compared with the leading states, the lagging states may have worse MI outcomes (e.g., higher MI mortality, incidence, and prevalence, lower survival and remaining LE), and the three scenarios (i.e., pre-existing prevalence, incidence, survival) may all contribute to the MI mortality disparities. Findings of the study will provide evidence for health professionals, researchers and policy decision-makers to devise and implement interventions and health policies to reduce the geographic disparities of MI, and ultimately mitigate the LE gap across the U.S.

## Materials and Methods

### Data Source

Two data sources covering the 2000–2017 period were used in the study. First, data on death certificate-based mortality (CBM) were directly derived from the Wide-Ranging Online Data for Epidemiologic Research (WONDER) of the U.S. Center for Disease Control and Prevention (CDC) (18). Second, data on MI incidence-based mortality (IBM), incidence, prevalence, prevalence at age 65, survival after MI diagnosis, and remaining LE at age 65 among MI patients were derived from a 5% sample of over five million Medicare beneficiaries (both Part A and Part B) (19). Individuals whose Medicare coverage was <20% of their months were excluded. Patients who were coded as 410 and 411 in the International Classification of Disease, Ninth Revision (ICD-9), and I21–I22 and I24 in the ICD-10 were categorized as MI. The Medicare provides a good data source that is from a national representative sample of older adults aged 65+, covers the whole geographic regions in the U.S., and includes both morbidity and mortality indicators, enabling us to examine the geographic disparities of MI outcomes.

### Ethics Approval

This is a secondary data analysis. All data analyses were designed and performed in accordance with the ethical standards of the responsible committee on human studies and with the Declaration of Helsinki (of 1975, revised in 2013), and have been approved by the Duke University Health System Institutional Review Board for Clinical Investigations (IRB FWA00009025).

### Leading and Lagging States

To characterize the geographic disparities, the leading (i.e., Hawaii, Florida, Arizona, Connecticut, Minnesota, and Colorado, California and New York) and lagging (i.e., Arkansas, Tennessee, Louisiana, Oklahoma, Kentucky, Alabama, Mississippi, and West Virginia) states were selected based on the LE at age 65 (17). Among the leading states, New York and California were excluded from the analysis due to their higher heterogeneity in healthcare service and primarily urban regions.

### Variable Measures

Death certificate-based mortality (CBM) from MI as the underlying cause of death (CBM-UCD) was computed based on the number of deaths immediately caused by MI, while CBM from MI as multiple cause of death (CBM-MCD) was computed based on the number of deaths by any causes with MI being one comorbidity. IBM was computed based on the number of all-cause deaths occurring in individuals with a prior MI diagnosis. MI incidence was defined based on the date of the earliest record with a primary diagnosis of MI if a second confirmatory record of MI appeared no later than 0.3 years afterwards (20). MI prevalence was defined based on an individual's diagnosis record of MI during a 12-month lookback period. MI prevalence at age 65 was estimated as the number of people with MI at age 65 divided by the respective total number of people at age 65 when they were enrolled in the Medicare program. One, 3 and 5-year survival rates were defined based on the date of death available in the Medicare records. Remaining LE at age 65 among MI patients were calculated based on the Medicare records of MI onset and death (21). Detailed computation of these variables can be found in the Supplementary Methods.

### Statistical Analysis

The characteristics of the study sample can be found in Supplementary Table 1. The estimated CBMs, IBM, incidence, prevalence, prevalence at age 65, and survival rates after MI diagnosis and remaining LE at age 65 were age-standardized based on the U.S. 2000 standard population. The temporal trends of these MI outcomes were compared between the leading and lagging states. Sex- and race-specific trends were included in the Supplementary Materials (Supplementary Figures 1–5); 95% confidence intervals (CI) were used to compare the differences between leading and lagging states with no overlapping of the 95% CI indicating significant differences at *p*-value < 0.05.

To quantify the geographic disparities in MI incidence and survival after diagnosis, Cox proportional hazards model was used. The Cox models were analyzed for total sample and separately by sex- and race-specific groups, that was then further stratified by age groups (65–79 and 80+). In the Cox models for survival in the age group 80+, the analysis was further stratified by the age of diagnosis (65–79 and 80+). The Cox estimates for White and Black were included in the main results, and more detailed race-specific analysis (i.e., Hispanics, Asian, Native American, and other races) was shown in the Supplementary Materials (Supplementary Figure 6). Age was controlled in the incidence Cox model, and age of diagnosis was controlled in the survival Cox model. In the total sample model, both sex and race were controlled, while in the sex- and race-specific models, race and sex were controlled, respectively. The software SAS was used for all statistical analyses (Version 9.4, SAS Institute, Inc., Cary, NC).

## Results

### Characteristics of the Study Sample

In the leading states, the proportion of males did not change (ranging from 42.2% in 2000 to 45.6% in 2017), while the proportion of White and Black populations varied from 88.7% to 85.7%, and from 3.6% to 4.4% during the same period, respectively. In the lagging states, the proportion of males increased from 39.8% in 2000 to 44.5% in 2017, while the respective proportion for White and Black ranged from 84.5% to 84.1%, and from 12.0% to 11.5% (Supplementary Table 1).

### Certificate-Based Mortality

**Figure 1:**
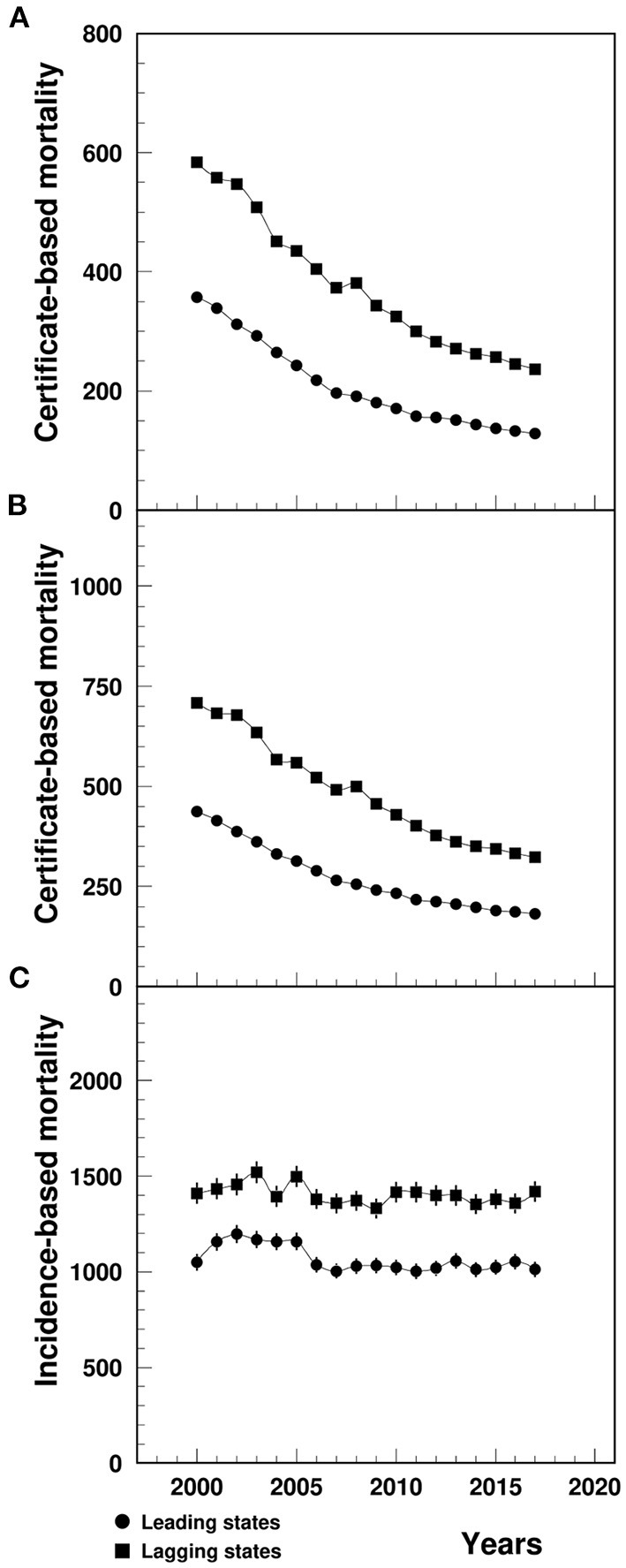
Temporal trend of CBM-UCD **(A)**, CBM-MCD **(B)** and IBM **(C)** (1/100,000) of MI among patients aged 65+ in the U.S. leading and lagging states. CBM-UCD, Certificate-based mortality from MI as underlying cause of death, CBM-MCD, Certificate-based mortality from MI as the multiple cause of death, IBM, Incidence-based mortality, MI, Myocardial infarction. Note: ^1^Data for **(A,B)** were derived from CDC WONDER, and data for **(C)** were derived from 5% Medicare Beneficiaries. The 95%CI were too small to show up in the CBM plots **(A,B)**.

The MI CBM-UCD (1/100,000) in the lagging states declined from 583.7 in 2000 to 236.7 in 2017, significantly higher than that in the leading states, which ranged from 357.6 to 128.2 during the same study period (Figure 1A). The CBM-MCD (1/100,000) in the lagging states declined from 707.7 in 2000 to 322.7 in 2017, significantly higher than that in the leading states which ranged from 437.7 to 182.4 (Figure 1B). The between-the-state difference was narrowing with time.

### Incidence-Based Mortality

The MI IBM (1/100,000) in the lagging states varied from 1409.6 in 2000 to 1419.1 in 2017, significantly higher than that in the leading states ranged from 1050.4 in 2000 to 1012.1 in 2017 (Figure 1C).

### MI Incidence

**Figure 2:**
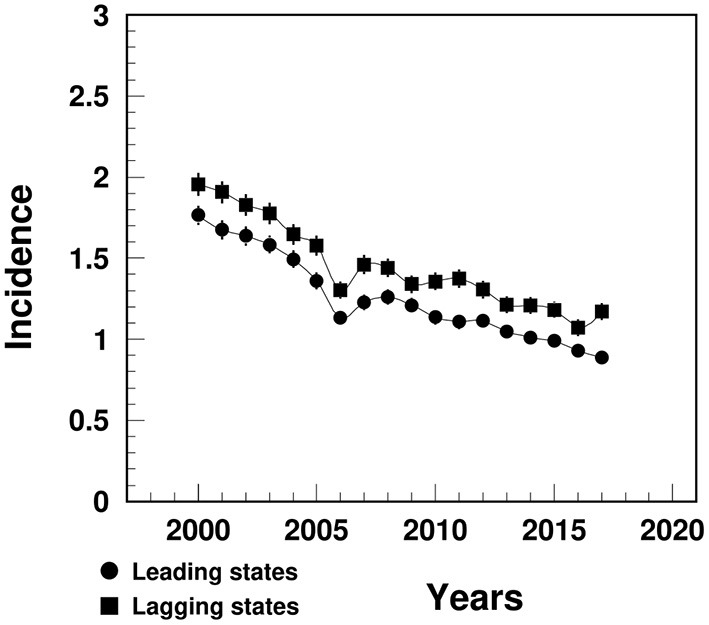
Temporal trend of MI incidence (%) among people aged 65+ in the U.S. leading and lagging states. Note: ^1^Data were derived from 5% Medicare Beneficiaries. ^2^The sudden decline in 2005–2006 that mainly occurred among females could potential be contributed from the Medicare Policy change (https://www.liebertpub.com/doi/10.1089/jwh.2012.3777).

The MI incidence in the lagging states declined from 2.0% in 2000 to 1.2% in 2017, significantly higher than that in the leading states (varying from 1.8 to 0.9%) (Figure 2).

### MI Prevalence

**Figure 3:**
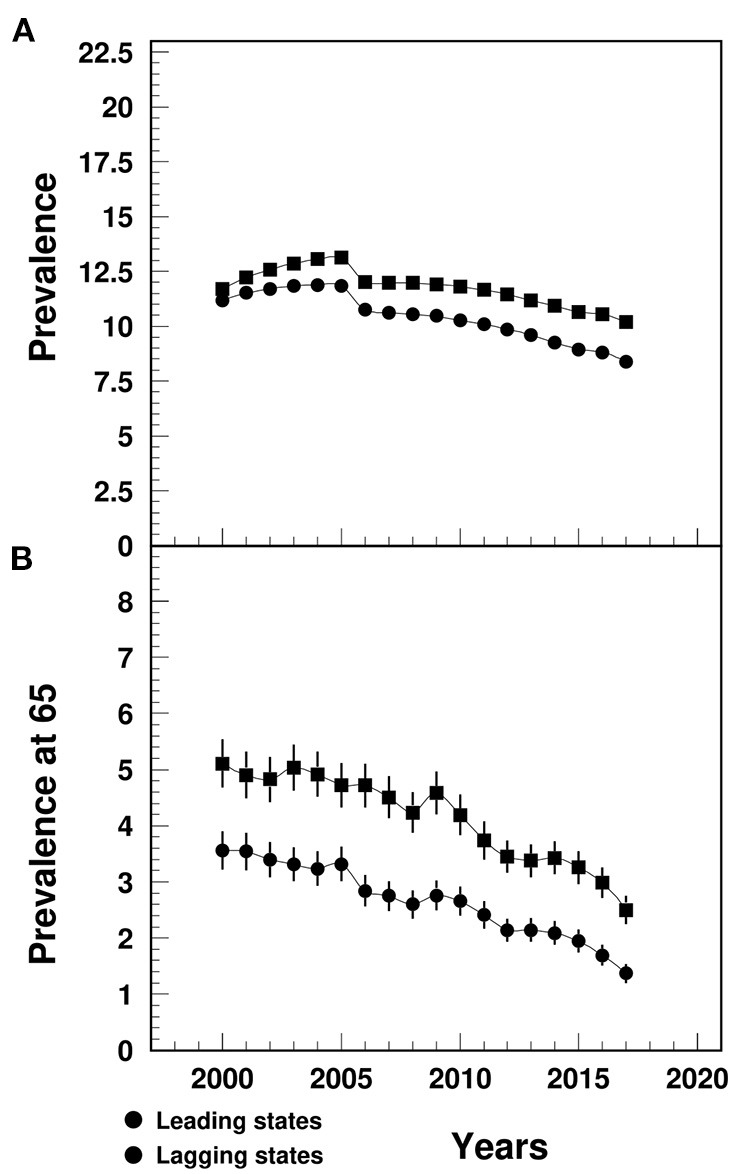
Temporal trend of MI prevalence **(A)** among people aged 65+ and prevalence at age 65 **(B)** (%) in the U.S. leading and lagging states. Note: ^1^Data were derived from 5% Medicare Beneficiaries. ^2^The sudden decline in 2005–2006 that mainly occurred among females could potential be contributed from the Medicare Policy change (https://www.liebertpub.com/doi/10.1089/jwh.2012.3777). ^3^The 95%CI bars in **(A)** were too small to show up in the plot.

The MI prevalence in the lagging states increased from 11.7% in 2000 to 13.1% in 2005, followed by a decline to 10.2% in 2017, significantly higher than the similar trend in the leading states (varying from 11.2 to 8.4%) (Figure 3A). The between-the-state difference was widening over time.

The MI prevalence at age 65 in the lagging states declined from 5.1% in 2000 to 2.5% in 2017, that was significantly higher than the leading states (ranging from 3.6 to 1.4%) (Figure 3B). The between-the-state difference declined since 2009.

### Survival After MI Diagnosis

**Figure 4:**
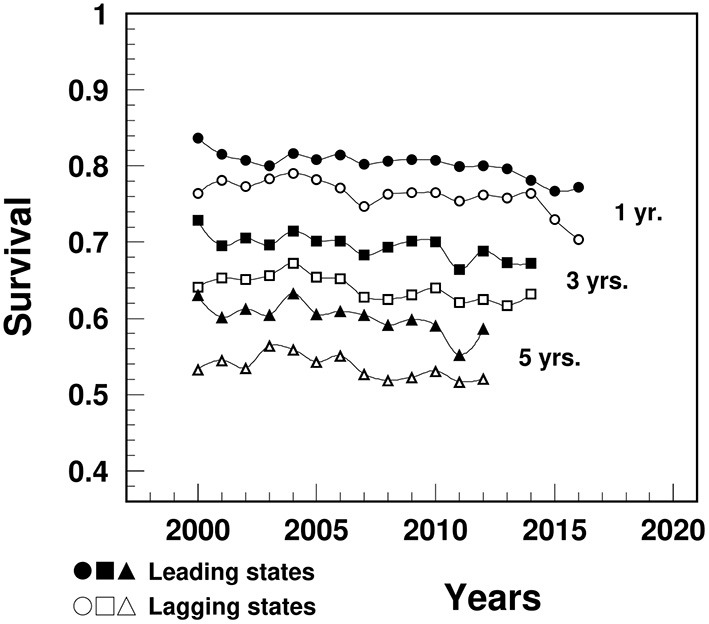
Temporal trend of 1-, 3-, and 5-year survival after a MI diagnosis among people aged 65+ in the U.S. leading and lagging states. Note: Data were derived from 5% sample of Medicare Beneficiaries.

The survival rates after a MI diagnosis in the lagging states were significantly lower compared to the leading states for 1-year (varying from 76.4% in 2000 to 70.4% in 2016 vs. 83.7 to 77.2%), 3-year (from 64.1% in 2000 to 63.2% in 2014 vs. 72.9 to 67.2%) and 5-year survival (from 53.3% in 2000 to 52.1% in 2012 vs. 63.1 to 58.7%) (Figure 4).

### Life Expectancy at Age 65

**Figure 5:**
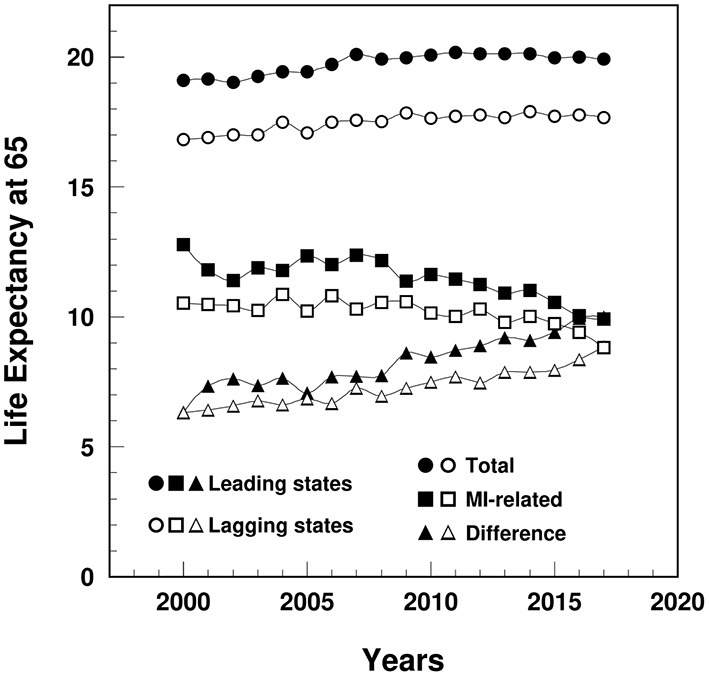
Temporal trend of remaining life expectancy at age 65 (years) among MI patients and non-MI patients and their gap in the leading and lagging states. Note: Data were derived from 5% sample of Medicare Beneficiaries.

The remaining LE at age 65 (years) among MI patients in the lagging states decreased from 10.5 in 2000 to 8.9 in 2017, significantly lower than the leading states (ranged from 12.8 to 9.9) (Figure 5). The remaining LE gap between MI and non-MI patients widened in the lagging states from 6.3 in 2000 to 8.9 in 2017, narrower than the gap in the leading states (from 6.3 to 10.0).

### Effects of Residence in the Lagging States

**Figure 6:**
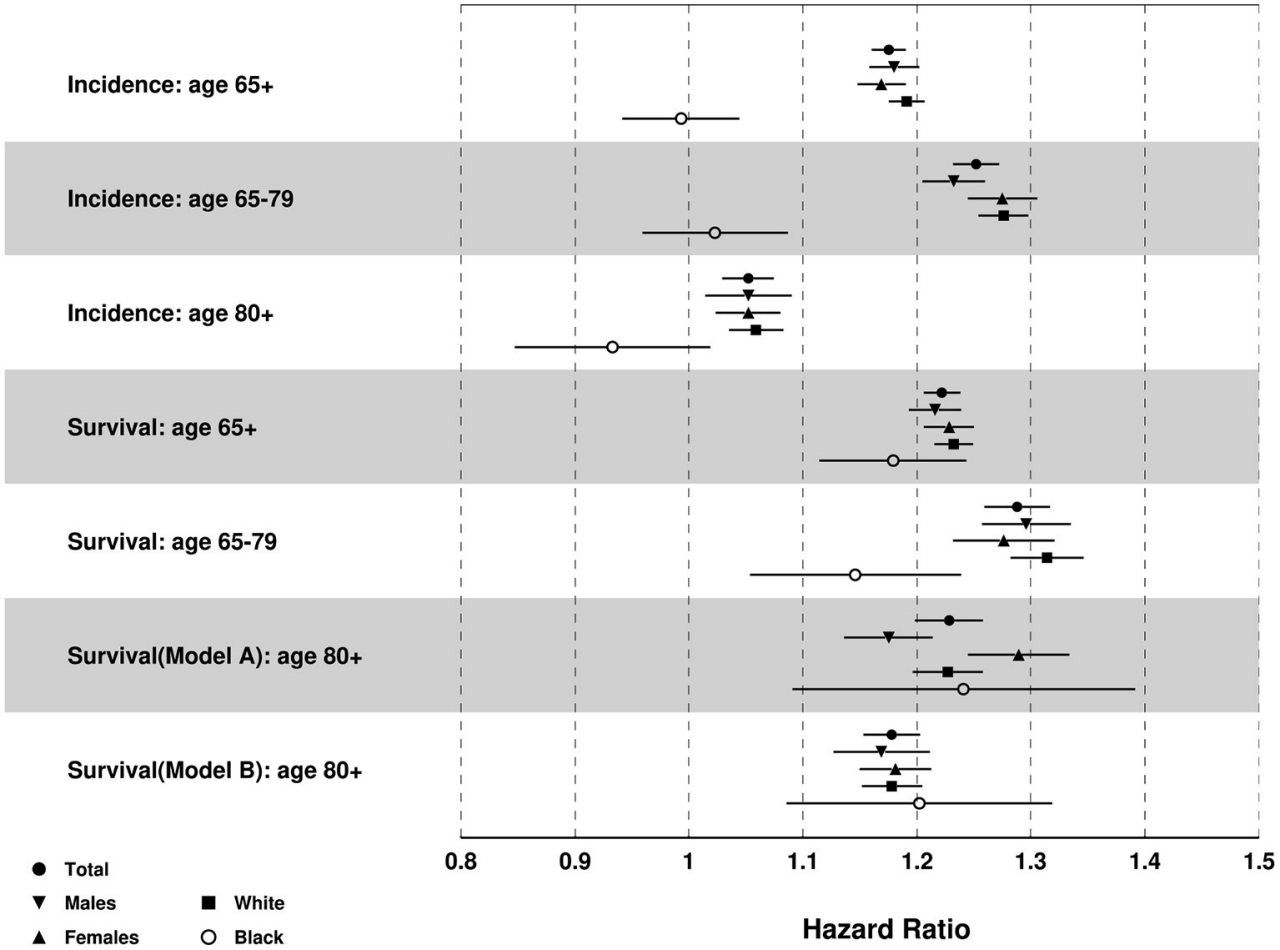
Results of multivariate Cox proportional hazards regression for MI incidence and death after MI diagnosis: Adjusted hazards ratio (HR) [95% CI] of lagging vs. leading states. Note: Age was controlled for incidence, and age of diagnosis was controlled for survival after MI diagnosis.

Results from Cox model (Figure 6) showed that old adults living in the lagging states had greater risk of MI incidence than their counterparts from the leading states [Adjusted Hazards Ratio, AHR (95% Confidence Interval, CI)]: 1.18 (1.16, 1.19) for total sample, 1.18 (1.16, 1.20) for males, 1.17 (1.15, 1.19) for females, 1.19 (1.18, 1.21) for White, but not for Blacks 0.99 (0.94, 1.05). Compared with people aged 80+, individuals aged <80 had more pronounced between-the-state differences in the risk of MI incidence [1.25 (1.23, 1.27) vs. 1.05 (1.03, 1.08) for total sample].

Results in Figure 6 also showed that old adults in the lagging states had greater risk of death after MI diagnosis [AHR (95%CI)]: 1.22 (1.21, 1.24) for total sample, 1.22 (1.19, 1.24) for males, 1.23 (1.21, 1.25) for females, 1.23 (1.22, 1.25) for White, and 1.18 (1.12, 1.25) for Black population. Individuals aged <80 also showed greater between-the-state differences in the risk of death after MI diagnosis than those aged 80+: 1.29 (1.26, 1.32) for individuals aged 65–79 years old, 1.23 (1.20, 1.26) for individuals aged 80+ and age of MI diagnosis <80, and 1.18 (1.15, 1.20) for individuals age 80+ and age of MI diagnosis 80+.

Cox model results for other races showed similar results with the total sample except that Hispanics in the lagging states had lower incidence than the leading states [0.71 (0.56, 0.89)] (Supplementary Figure 6).

## Discussion

This study found that compared with the states leading by LE, lagging states had substantially higher MI mortality, incidence, prevalence, as well as lower survival and remaining LE at age 65. Most of the mortality disparities were due to the between-the-state differences in MI incidence, pre-existing prevalence at age 65, and survival. Higher MI mortality rates in the lagging states are consistent with previous studies showing the U.S. East and West South Central regions having higher mortality from MI (22, 23). In our study, all three scenarios (incidence, survival, and pre-existing prevalence) contributed substantially to the geographic disparities in MI mortality. Higher MI incidence rates in the lagging states are consistent with other studies (22) and may be attributable to the higher prevalence of risk factors including, but not limited to, hypertension, stroke, angina, diabetes and mental health problems (24), obesity (25), cigarettes smoking (26), and physical inactivity (27), with the impacts of these risk factors on MI incidence varying by geographic region (28–30). The prevalence of MI at age 65 (when older U.S. adults are enrolled in the Medicare program), was also higher in the lagging states and suggested early onset of MI in the lagging states. It is consistent with data from the Behavioral Risk Factor Surveillance System (BRFSS) studies that showed higher prevalence of MI at ages younger than 65 in the lagging states (31) potentially associated with higher prevalence of MI risk factors in the lagging states among younger adults (24–27, 32). Finally, observed lower survival rates after MI diagnosis and lower remaining LE at age 65 among MI patients may be explained by earlier MI onset (31), lower rate of cardiac rehabilitation (33), worse nursing home performance (34), lower adherence to medication intake for arterial hypertension and cholesterol-lowering (35), and longer pre-hospital time to the percutaneous coronary intervention (PCI) centers (36) in the lagging states.

Higher MI mortality among males and in the Black population could be associated with lower survival in these two groups (8), while greater geographic disparities in MI incidence among Whites than Blacks could be associated with greater race-specific between-the-state differences in the prevalence of risk factors (37). Higher incidence of MI in the leading states could be due to the higher prevalence of risk factors among Hispanics in the leading states (37, 38) or the possible underdiagnosis of MI in the lagging states, that is attributable to the lower access to health care and lower coverage of health insurance among Hispanics in these LE lagging states (39).

Older adults aged 65–79 years had more pronounced between-the-state differences in MI incidence than people aged 80+. This can be explained by the fact that people with MI risk factors may not be able to survive to age 80, thus leading to greater between-the-state differences in prevalence of risk factors in people aged 65–79 (31). It is also likely that more MI patients aged 65–79 would die in the lagging states compared to the leading states. Thus, after passage of high-risk MI patients before age 80 (more cases died in the lagging states), the remaining MI patients in both states may show less geographic differences in the risk of death (40).

Both leading and lagging states had consistently declining trends of MI mortality (i.e., CBMs) that were consistent with some other studies (11, 24), while IBM for MI entered a plateau stage in recent years. The decade-long declines in CBMs may be attributable to the advances and improvements in MI and other heart diseases treatment and management [e.g., the emergency reperfusion of ischemic myocardium (41), PCI treatment (14), enhancements in timeliness of emergency medical systems (42)].

Compared with the data from death certificates (i.e., CBMs), IBM, estimated based on administrative Medicare data, may be more sensitive to earlier detection of the change of mortality since it contains additional information regarding the disease morbidity. The recent decade-long plateau stage in IBM may be mainly attributed to the dynamics in incidence and survival. The decade-long declines in incidence, consistent with previous studies (24, 43), may be attributable to the improvements in primary prevention efforts, including lifestyle alternation and pharmacological interventions (44), and improved awareness, treatment and control of cardiovascular disease risk factors (45, 46). We also observed a sudden dip in MI incidence and prevalence during 2005–2006 among females, that may be related to the “Welcome to Medicare” visit (WMV) effective on January 1, 2005 (47). In the WMV, women will receive additional breast and cervical cancer screening test. Thus, more females would join the Medicare program, leading to a greater total female population with smaller incidence and prevalence. Another reason may be the introduction of Medicare Part D in 2006 where more females were enrolled than males (48).

One alarming finding of the study was the recent declines in survival, which was also corresponding to the declines in remaining LE among MI patients. The declining survival may be attributed to, at least in part, the reduced length of hospital stay (49, 50), that followed by less-than-optimal self-management of MI (51) and inadequate post-discharge management of the increasing MI complications and comorbidities (50, 52, 53), as well as the increases in mortalities from non-cardiovascular diseases after hospital discharge among older MI patients (16).

Another potential cause of the declining survival is the Hospital Readmissions Reduction Program (HRRP) that aims to encourage hospitals to improve the quality of health care, and in turn, to reduce the readmission rates. HRRP was discussed during 2007–2009, announced in 2010 and implemented in 2012 that imposes Medicare payment penalties on hospitals with higher-than-expected readmission rate (54). Three diseases were initially covered in HRRP, including acute MI, heart failure and pneumonia. However, previous studies indicated that MI mortality did not significantly increase corresponding to the HRRP (55–58). Since mortality contains information from multiple components (e.g., incidence, survival), it is not as sensitive as survival to reflect the change as shown in this study. If the causal relationship between the HRRP and declining survival can be confirmed, urgent actions are needed to amend the respective policy.

### Limitations

Due to the unavailability of Medicare data for detailed subtypes of MI [e.g., STEMI, non-ST elevation MI (NSTEMI)], cautions are needed when generalizing the geographic differences and temporal trend of MI overall shown in the study to individual subtypes of MI. Electronic health records with more detailed MI subtype information will be used in the future studies to differentiate the distinct pattern of geographic disparities and temporal trends of MI subtypes, and to identify the core subtype to be intervened.

## Conclusion

There are substantial geographic disparities in MI outcomes across the U.S. with the lagging states having higher mortality, incidence, and prevalence, lower survival and remaining LE among older adults. The disparities in MI mortality were mainly attributable to the between-the-state differences in MI incidence, pre-existing prevalence at age 65, and survival after MI diagnosis. We also observed alarming declining trends in survival and remaining LE among MI patients that may suggest the potential increase in mortality, underscoring the urgent need of investigation of contributing factors that must be addressed.

## Data Availability Statement

The data analyzed in this study is subject to the following licenses/restrictions: Data were obtained from Centers for Medicare & Medicaid Services that contain personally identifiable information under a data use agreement. These data are currently stored on a secure server at the Duke University. Requests to access these datasets should be directed to Centers for Medicare & Medicaid Services (https://www.cms.gov/).

## Ethics Statement

The studies involving human participants were reviewed and approved by Duke University Health System Institutional Review Board. Written informed consent for participation was not required for this study in accordance with the national legislation and the institutional requirements.

## Author Contributions

BY, IA, and JK contributed to the conception and design of the study. IA performed the statistical analyses. BY wrote the first draft of the manuscript. IA, AY, and JK reviewed and improved the manuscript. All authors read and approved the submitted version.

## Funding

The work was supported by the National Institute on Aging (#: R01-AG057801). The sponsors had no role in the design and conduct of this study.

## Conflict of Interest

The authors declare that the research was conducted in the absence of any commercial or financial relationships that could be construed as a potential conflict of interest.

## Publisher's Note

All claims expressed in this article are solely those of the authors and do not necessarily represent those of their affiliated organizations, or those of the publisher, the editors and the reviewers. Any product that may be evaluated in this article, or claim that may be made by its manufacturer, is not guaranteed or endorsed by the publisher.
