# Supplementary material for: Epidemiology of Geographic Disparities of Myocardial Infarction Among Older Adults in the United States: Analysis of 2000–2017 Medicare Data
Source: Front Cardiovasc Med. 2021 Sep 9;8:707102. doi: 10.3389/fcvm.2021.707102 (PMC8458897; doi:10.3389/fcvm.2021.707102)
Supplement: Supplementary file 1 [file Data_Sheet_1.pdf]

Supplement

eTable 1. Characteristics of the study sample, n (%)

Variable	2000	2002	2004	2006	2008	2010	2012	2014	2016	2017
Leading states										
**Sex**										
Male	92376 (42.17)	97219 (42.58)	100486 (42.91)	104308 (39.58)	104888 (40.56)	107498 (41.33)	111571 (42.31)	116829 (42.92)	117300 (44.94)	120204 (45.55)
Female	126702 (57.83)	131104 (57.42)	133684 (57.09)	159196 (60.42)	153730 (59.44)	152625 (58.67)	152104 (57.69)	155404 (57.08)	143688 (55.06)	143678 (54.45)
**Race**										
White	194322 (88.70)	202072 (88.50)	206738 (88.29)	232716 (88.32)	228164 (88.22)	228483 (87.84)	230532 (87.43)	235936 (86.67)	224366 (85.97)	226046 (85.66)
Black	7869 (3.59)	8644 (3.79)	9265 (3.96)	10612 (4.03)	10429 (4.03)	10904 (4.19)	11072 (4.20)	11691 (4.29)	11302 (4.33)	11487 (4.35)
Hispanic	5052 (2.31)	5271 (2.31)	5386 (2.30)	6109 (2.32)	6007 (2.32)	6259 (2.41)	6206 (2.35)	6341 (2.33)	5782 (2.22)	5634 (2.14)
Asian	3479 (1.59)	3641 (1.59)	3724 (1.59)	4198 (1.59)	4179 (1.62)	4217 (1.62)	4303 (1.63)	4557 (1.67)	4569 (1.75)	4695 (1.78)
Native American	817 (0.37)	917 (0.40)	970 (0.41)	1112 (0.42)	1137 (0.44)	1228 (0.47)	1325 (0.50)	1467 (0.54)	1549 (0.59)	1609 (0.61)
Others	7089 (3.24)	7397 (3.24)	7772 (3.32)	8446 (3.21)	8405 (3.25)	8333 (3.20)	8267 (3.14)	8405 (3.09)	7962 (3.05)	8147 (3.09)
Unknown	450 (0.21)	381 (0.17)	315 (0.13)	311 (0.12)	297 (0.11)	699 (0.27)	1970 (0.75)	3836 (1.41)	5458 (2.09)	6264 (2.37)
Lagging states										
**Sex**										
Male	73956 (39.79)	75527 (40.16)	77291 (40.73)	79965 (37.71)	78777 (38.73)	79806 (39.49)	83078 (40.61)	86251 (41.55)	87230 (43.78)	89601 (44.47)

Female	111923 (60.21)	112550 (59.84)	112465 (59.27)	132105 (62.29)	124617 (61.27)	122262 (60.51)	121485 (59.39)	121353 (58.45)	111995 (56.22)	111871 (55.53)
**Race**										
White	156995 (84.46)	159228 (84.66)	160874 (84.78)	180568 (85.15)	173736 (85.42)	172310 (85.27)	174112 (85.11)	175513 (84.54)	167957 (84.31)	169377 (84.07)
Black	22223 (11.96)	22028 (11.71)	21905 (11.54)	23981 (11.31)	22428 (11.03)	22507 (11.14)	22823 (11.16)	23693 (11.41)	22619 (11.35)	23148 (11.49)
Hispanic	306 (0.16)	343 (0.18)	359 (0.19)	415 (0.20)	434 (0.21)	477 (0.24)	512 (0.25)	602 (0.29)	695 (0.35)	733 (0.36)
Asian	437 (0.24)	497 (0.26)	555 (0.29)	673 (0.32)	702 (0.35)	789 (0.39)	841 (0.41)	944 (0.45)	984 (0.49)	1029 (0.51)
Native American	1345 (0.72)	1532 (0.81)	1616 (0.85)	1925 (0.91)	1910 (0.94)	1923 (0.95)	2011 (0.98)	2129 (1.03)	2089 (1.05)	2120 (1.05)
Others	4148 (2.23)	4121 (2.19)	4180 (2.20)	4242 (2.00)	3972 (1.95)	3730 (1.85)	3519 (1.72)	3305 (1.59)	2985 (1.50)	2892 (1.44)
Unknown	425 (0.23)	328 (0.17)	267 (0.14)	266 (0.13)	212 (0.10)	332 (0.16)	745 (0.36)	1418 (0.68)	1896 (0.95)	2173 (1.08)

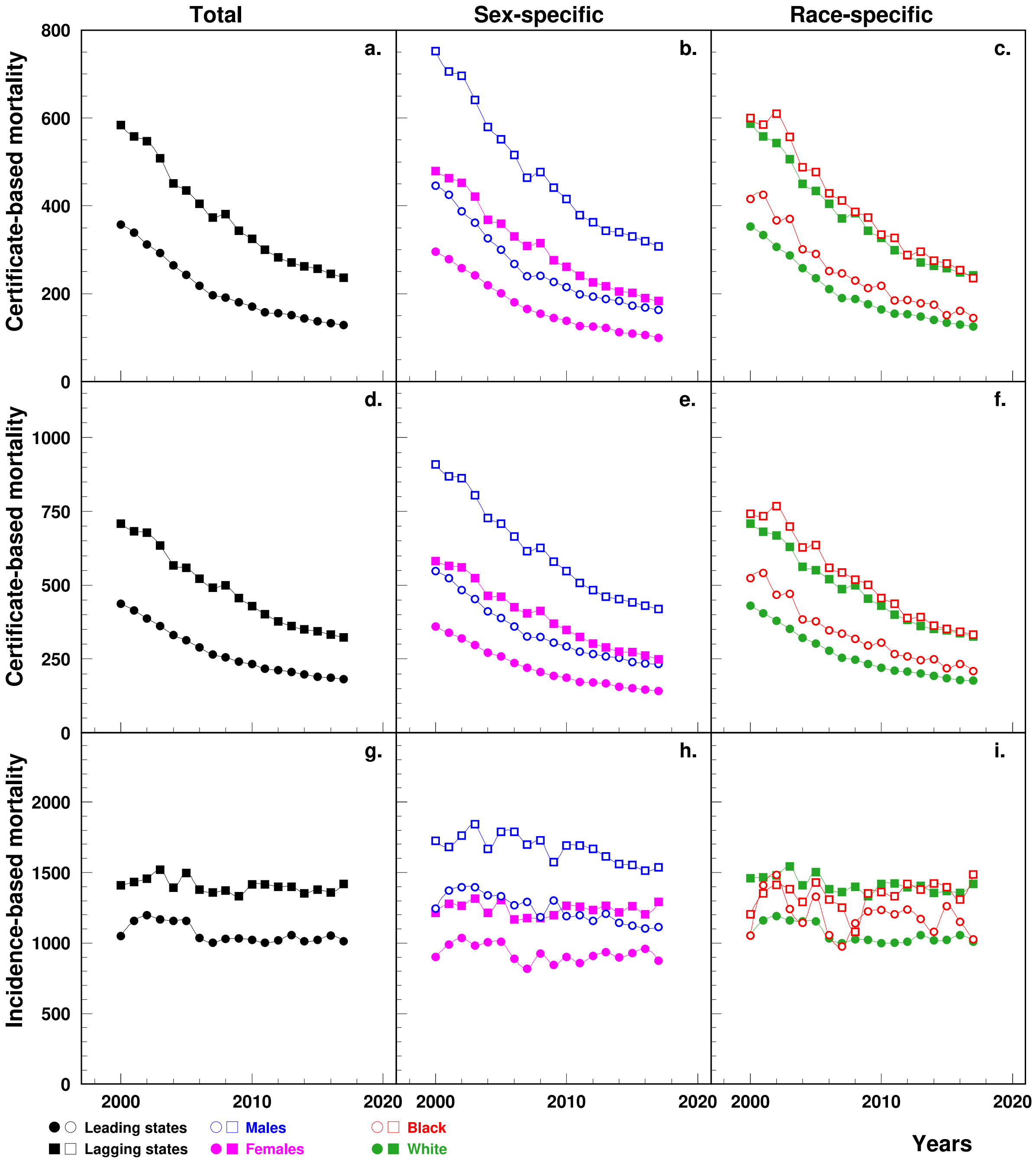

eFigure 1. Temporal trend of CBM-UCD (a-c), CBM-MCD (d-f) and IBM (g-i) (1/100,000) of MI among patients aged 65+ in the U.S. leading and lagging states, overall, by sex- and race-specific groups

Note: CBM-UCD= Certificate-based mortality from MI as underlying cause of death, CBM-MCD=Certificate-based mortality from MI as the multiple cause of death, IBM=Incidence-based mortality, MI=Myocardial infarction. [1]Data for Figure 1a-f were derived from CDC WONDER, and data for Figure 1g-i were derived from 5% Medicare Beneficiaries.

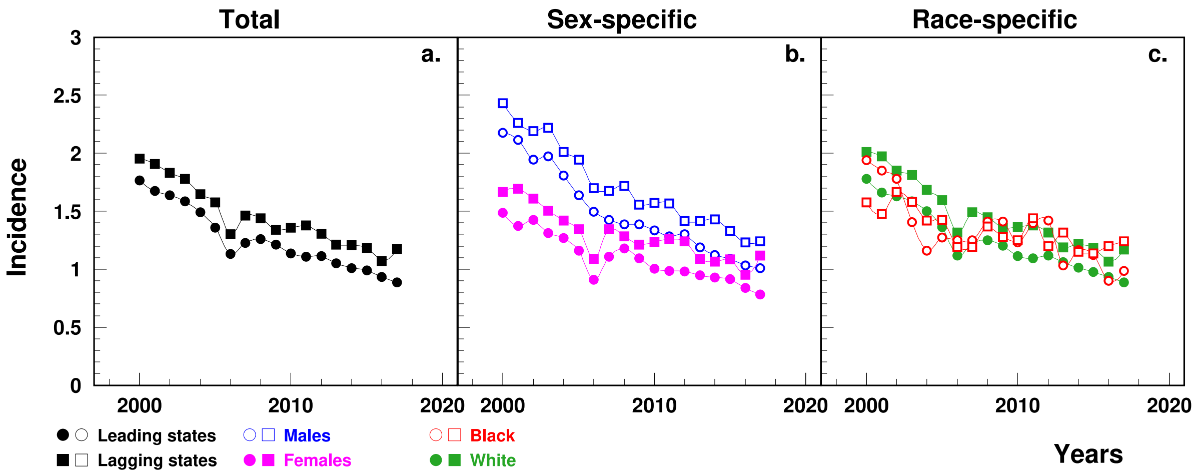

eFigure 2. Temporal trend of MI incidence (%) among people aged 65+ in the U.S. leading and lagging states, overall, by sex- and race-specific groups

Note: [1]Data were derived from 5% Medicare Beneficiaries. [2]The sudden decline in 2005-2006 that mainly occurred among females was associated with the Medicare Policy change (https://www.liebertpub.com/doi/10.1089/jwh.2012.3777).

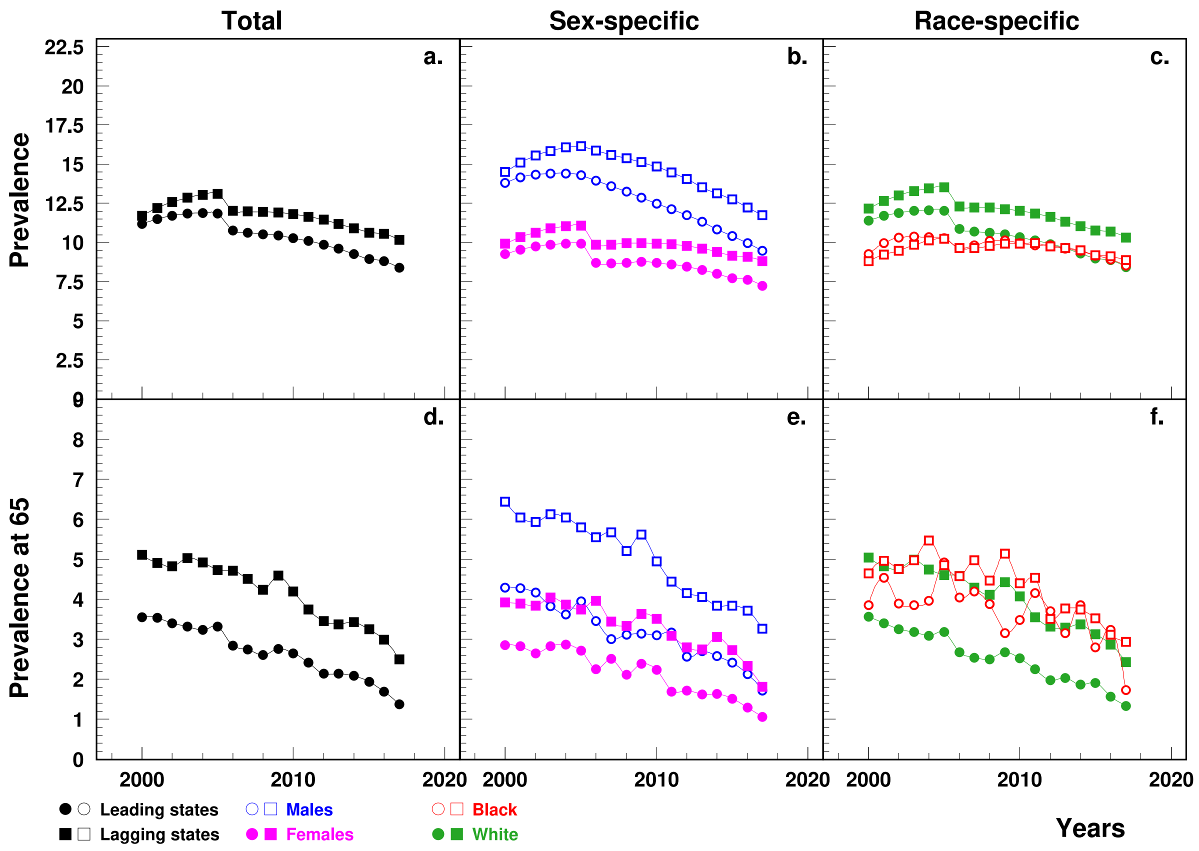

eFigure 3. Temporal trend of MI prevalence among people aged 65+ and prevalence at age 65 (%) in the U.S. leading and lagging states, overall, by sex- and race-specific groups

Note: [1]Data were derived from 5% Medicare Beneficiaries. [2]The sudden decline in 2005-2006 that mainly occurred among females was associated with the Medicare Policy change (https://www.liebertpub.com/doi/10.1089/jwh.2012.3777).

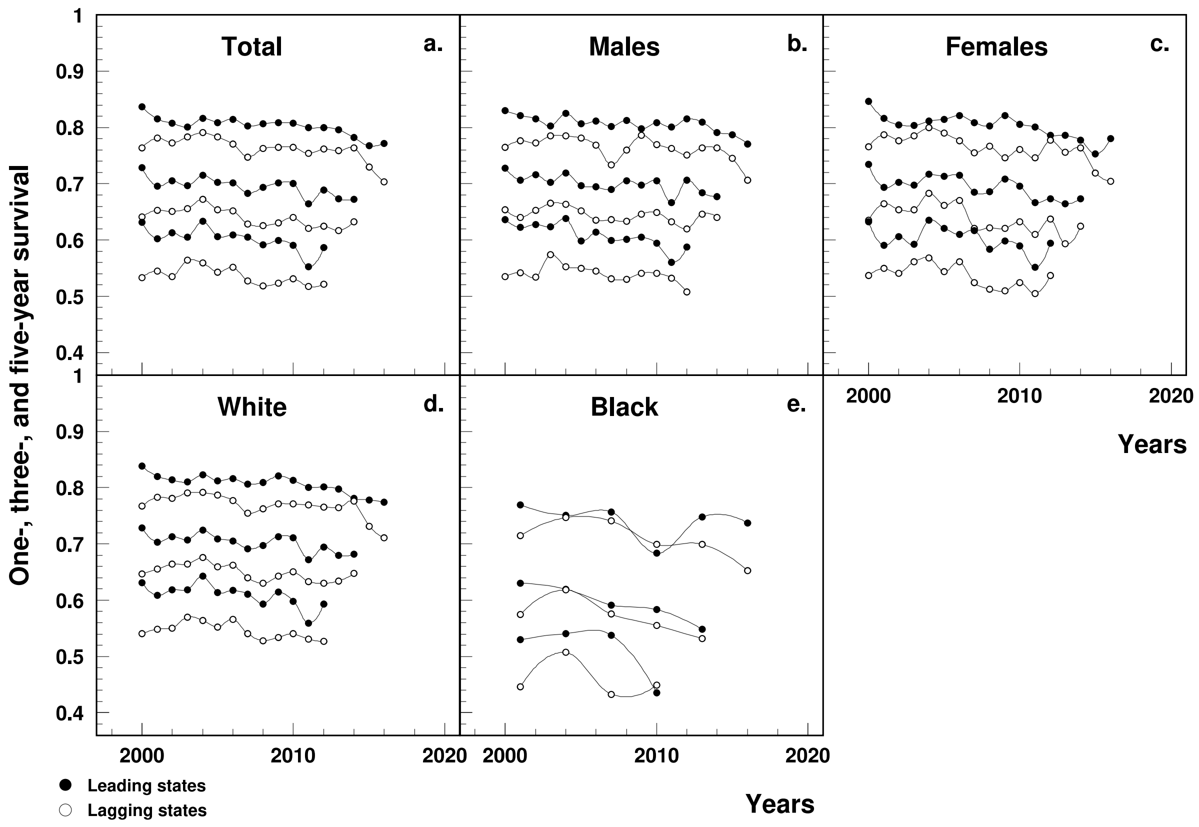

eFigure 4. Temporal trend of 1-, 3-, and 5-year survival after a MI diagnosis among people aged 65+ in the U.S. leading and lagging states, overall, by sex- and race-specific groups

Note: Data were derived from 5% sample of Medicare Beneficiaries. The survival curves for Blacks were smoothed by averaging the rates in consecutive three years to reduce the fluctuations that caused by the relatively small sample size.

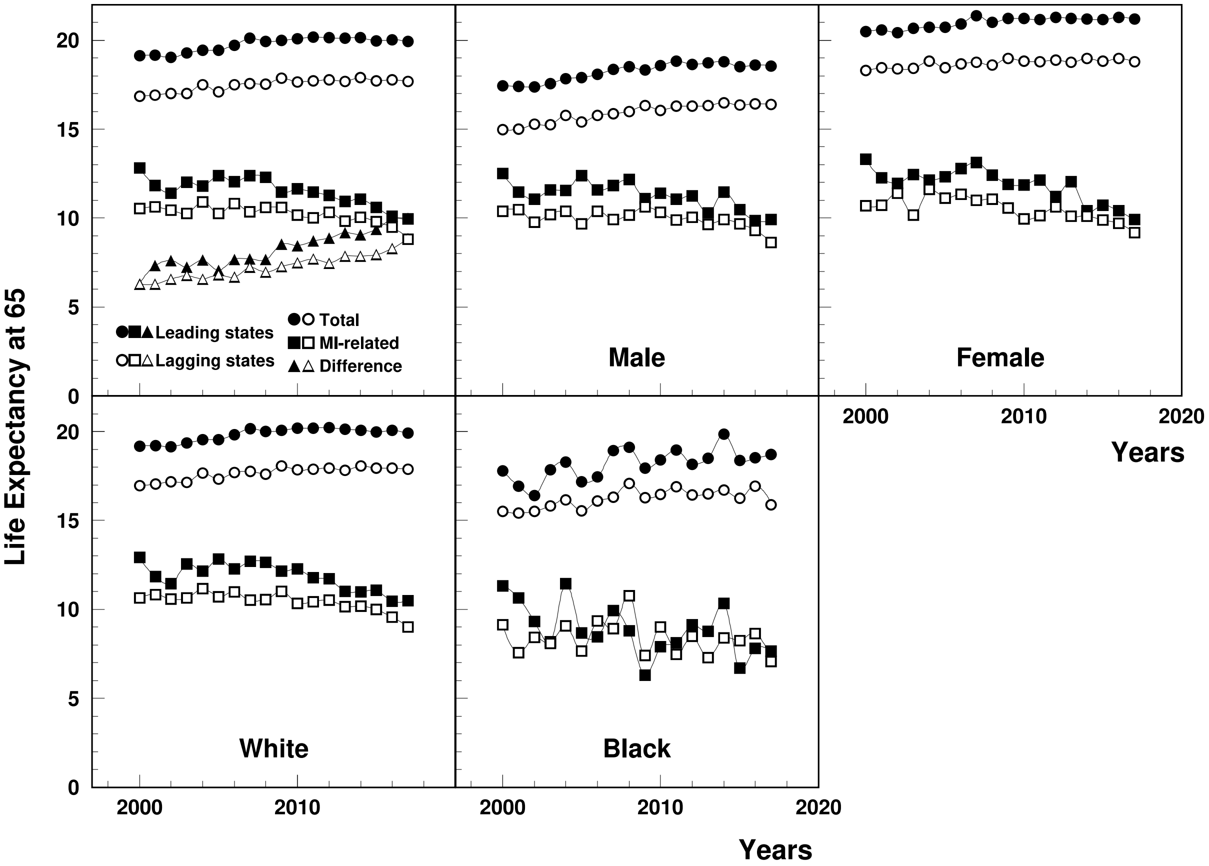

eFigure 5. Temporal trend of remaining life expectancy at age 65 (years) among MI patients and non-MI patients and their gap in the leading and lagging states, overall, by sex- and race-specific groups

Note: Data were derived from 5% sample of Medicare Beneficiaries.

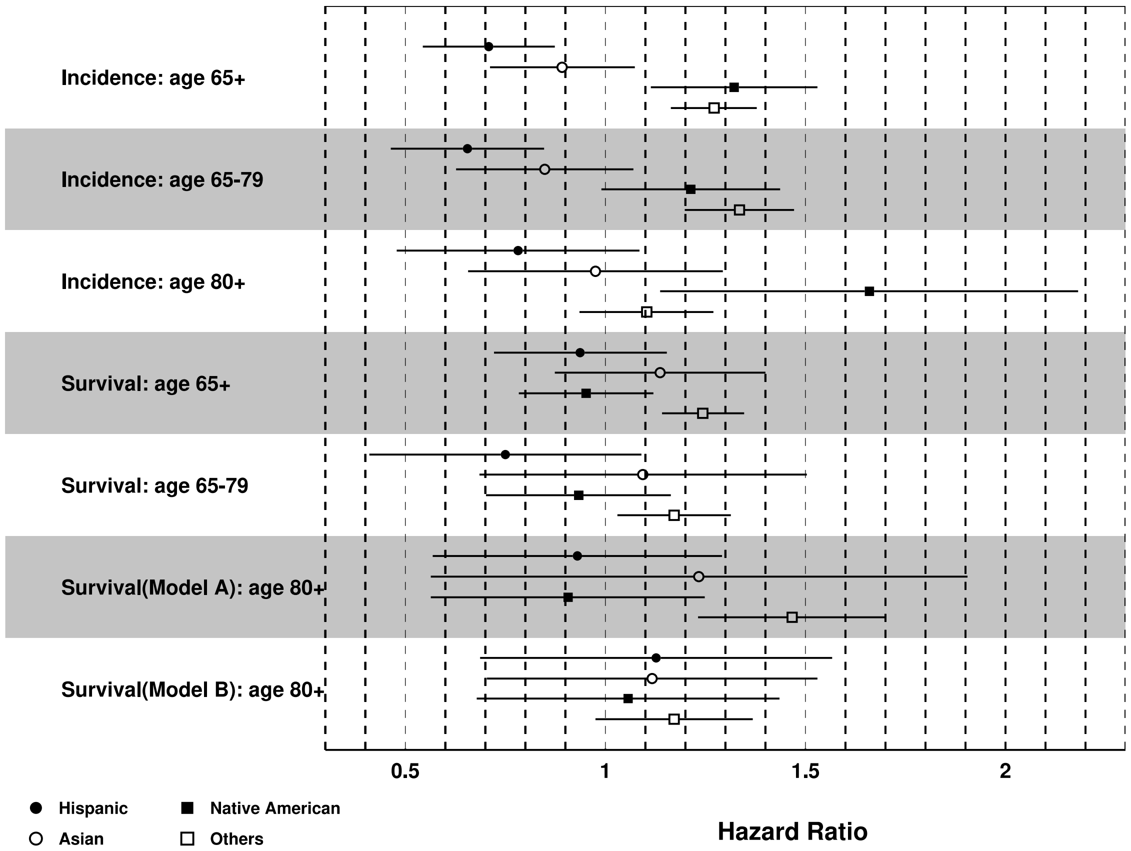

eFigure 6. Results of multivariate Cox proportional hazards regression for MI incidence and survival after MI diagnosis among Hispanics, Asian, Native Americans and other races: Adjusted hazards ratio (HR) [95% CI] of residence in the lagging states

Note: Age was controlled for incidence, and age of diagnosis was controlled for survival.

Supplementary Methods:

Medicare data and ascertainment algorithm [1] results in the following variables for each eligible individual: $a_{ini}^{(i)}$ — initial age of follow-up, $a_{fin}^{(i)}$ — final age of follow-up, $\delta_d^{(i)}$ — death/censoring indicator ($\delta_d^{(i)} = 1$ for death and $\delta_d^{(i)} = 0$ for censoring) at age $a_{fin}^{(i)}$; $a_{MI}^{(i)}$— age of MI diagnosis (or missing). The superscript (i) denotes individual characteristics. For each individual, dates of birth $y_b^{(i)}$ are available allowing us to calculate exact age at any point of the individuals' follow-up. An individual is considered prevalent at the time of Medicare enrollment if there exists a record with the MI code during 1 year after enrollment; $\delta_{p1}^{(i)}$ is an indicator of prevalence at the time of Medicare enrollment where $\delta_{p1}^{(i)} = 1$ for prevalent and $\delta_{p1}^{(i)} = 0$ for non-prevalent individuals; $\delta_{p0}^{(i)} = 1 - \delta_{p1}^{(i)}$ is an indicator of being non-prevalent at the time of Medicare enrollment.

We calculate prevalence, incidence rates, incidence-based mortality, and all-cause mortality in age- and time- bins (representing rectangles in the age and time plain where the Medicare data are available, eFigure 7). Specifically, the measures are calculated in 29 age groups including 25 one-year groups between ages 65 and 90, and three other groups: 90-91, 92-94, 95-99, 100-110. Person-years after age 110 are considered invalid and ignored. Similarly, we consider one-year calendar time groups in the period between year y_{ini} and y_{fin}. Thus, all age-specific measures are two-dimensional and calculated in age and time groups.

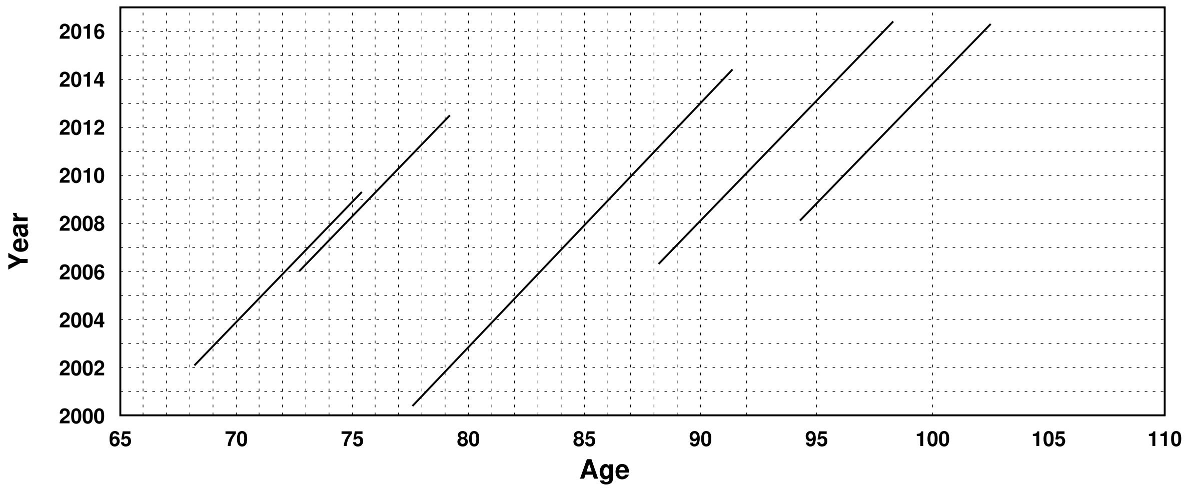

eFigure 7. Conceptual visualization of the age- and time- bins in Medicare data

An individual trajectory in this plain (also shown in eFigure 7) contributes to different rectangle bins during each individual follow-up period. Age-specific prevalence, incidence, incidence-based mortality, and all-cause mortality in a certain rectangle bin are calculated through the ratio N_c^{meas}/N_{py}, where N_c^{meas} is the number of effective cases that are measure specific (i.e., $meas = inc$ for incidence, $meas = prev$ for prevalence, $meas = mort$ for incidence-based mortality, and

$meas = allc$ for all-cause mortality). The number of person-years N_{py} is identical for all measures and calculated as:

$$N_{py} = \sum_i \left(y_2^{(i)} - y_1^{(i)}\right)$$

where $y_1^{(i)}$ and $y_2^{(i)}$ are the initial and final individual times for an age group with bounds defined by ages a_1 and a_2 in a time group with bounds y and $y+1$:

$$y_1^{(i)} = max\left(y - y_b^{(i)}, a_{ini}^{(i)}, a_1\right)$$
$$y_2^{(i)} = min\left(y + 1 - y_b^{(i)}, a_{fin}^{(i)}, a_2\right)$$

The numbers of effective cases are measure-specific:

$$N_c^{prev} = \sum_i \left(\delta_{p1}^{(i)}\left(y_2^{(i)} - y_1^{(i)}\right) + \delta_{p0}^{(i)} I\left(0 \le a_{MI}^{(i)} < y_2^{(i)}\right) max(\,0, y_2^{(i)} - max(\,a_{MI}^{(i)}, y_1^{(i)})\right)$$
$$N_c^{inc} = \sum_i \delta_{p0}^{(i)}\, I\left(y_1^{(i)} < a_{MI}^{(i)} \le y_2^{(i)}\right)$$
$$N_c^{mort} = \sum_i \delta_d^{(i)} I\left(y_1^{(i)} < a_{fin}^{(i)} \le y_2^{(i)}\right) I\left(0 \le a_{MI}^{(i)} < a_{fin}^{(i)}\right)$$
$$N_c^{allc} = \sum_i \delta_d^{(i)} I\left(y_1^{(i)} < a_{fin}^{(i)} \le y_2^{(i)}\right)$$

These formulas are illustrated in eFigure 8

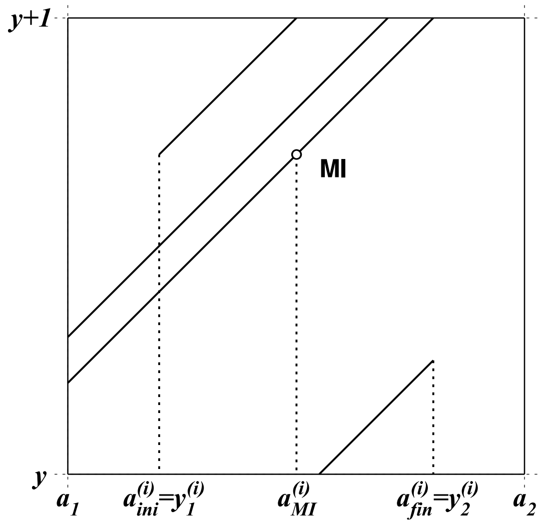

eFigure 8. Conceptual visualization of the formulas in the study

Reference

[1] Akushevich I, Kravchenko J, Ukraintseva S, Arbeev K, Yashin AI. Age Patterns of Incidence of Geriatric Disease in the U.S. Elderly Population: Medicare-Based Analysis. *Journal of the American Geriatrics Society*. 2012;60(2):323-327.
